# M. Velpeau on Flexions and Engorgement of the Uterus

**Published:** 1847-01

**Authors:** 


*M. Velpeau on Flexions and Engorgement of the Uterus.*— A proof of how often the term *engorgement* has expressed an error of diagnosis is found in the fact that of late years, and in proportion to the progress of science, engorgements of the uterus become more and more rare, while the number of vicious flexions is augmented. I do not fear to state, that of fifty women reputed to have engorgement, forty five will be found upon examination to be suffering from some deviation from the normal position of the organ. How can we explain this error of diagnosis being committed by well educated practitioners? The reason is simple. The woman is examined in the recumbent posture, and the finger meets, in a certain direction of the neck, forwards if there be anteflexion, and backward if there be retroflexion, with a tumor of considerable size and sensible, which is declared to result from an engorgement. But the tumor is simply the body of the organ bent at a more or less obtuse angle, and sometimes at a right angle. We can easily assure ourselves of this, especially when, as is the case with most women who have borne children, the walls of the abdomen are neither tense nor thick. By gently depressing the hypogastrium, we may grasp the body of the womb between the two hands, and appreciate its volume as accurately as if we could see it. Engorgement is one of the least usual conditions of the organ that we meet with, and on the contrary the body of the organ is often found somewhat atrophied.

Accurate diagnosis, in consequence of the treatment it designates, is here of great importance; for when we have to do with a simple deviation we dispense with the use of means proper for resolving a tumefaction which does not exist, of debilitating remedies each more mischievous than the other, and with confining the patient for months in the recumbent posture. We order for her, moderate exercise, a substantial and tonic regimen, antispasmodic, ferruginous drinks, saline baths, astringent vaginal injections, and lastly an abdominal bandage which may support the viscera, and prevent their weighing down the deviated organ.—*Gazette des Hopitaux.—Med. Chir. Rev.*

